# Understanding barriers and facilitators of inter-organizational dynamics in addressing substance use disorder among pregnant and parenting women

**DOI:** 10.1371/journal.pone.0336029

**Published:** 2025-11-12

**Authors:** Sugy Choi, Elizabeth Knopf, Megan A. O’Grady, Ivy Van Domselaar, Jessica Ortiz, Carla King, Charles J. Neighbors, Thomas D’Aunno

**Affiliations:** 1 Department of Population Health, New York University Grossman School of Medicine, New York, New York, United States of America; 2 Department of Public Health Sciences, University of Connecticut, UConn Health, Farmington, Connecticut, United States of America; 3 New York University Robert F. Wagner Graduate School of Public Service, New York, New York, United States of America; Virginia Mason Franciscan Health, UNITED STATES OF AMERICA

## Abstract

**Background:**

Pregnant and parenting women with substance use disorders (SUDs) face complex and overlapping challenges, including substance use, legal issues, housing instability, and trauma. Effective interorganizational collaboration is critical but often hindered by fragmented care and resource limitations. This study explores the key barriers and facilitators that impact collaborative efforts among healthcare providers, government agencies, and community organizations in addressing SUD among pregnant and parenting women.

**Methods:**

This qualitative study was conducted in New York State between April 2022 and April 2023. The study focused on organizations that provide services to pregnant and parenting women with SUDs, including government agencies, SUD treatment centers, healthcare settings, and community-based care organizations. Semi-structured, one-on-one interviews were conducted with staff to explore how their organizations coordinate care. Thematic analysis was used to identify patterns related to interorganizational collaboration. Primary data were collected through interviews with 30 staff members across multiple stakeholder groups: child welfare services (n = 8), criminal legal agencies (n = 5), health agencies (n = 3), healthcare service settings (n = 4), SUD treatment programs (n = 6), and community-based organizations (n = 4). Interviews lasted approximately one hour and focused on organizational roles, referral processes, and coordination efforts in serving the target population.

**Results:**

Collaborative care was primarily facilitated through referral networks, case management teams, and the presence of embedded healthcare professionals. However, these systems were frequently limited by fragmented communication, stigmatizing attitudes, and insufficient resources. Organizational facilitators included co-located healthcare staff within child welfare services and formalized partnerships across sectors. Key barriers included staffing shortages, burnout, and misalignment of organizational goals. At the individual level, collaboration often depended on informal relationships and staff-driven initiatives, though interdisciplinary knowledge gaps remained a significant challenge.

**Conclusions:**

Improving service coordination for pregnant and parenting women with SUDs will require stronger organizational infrastructure, investment in cross-sector communication strategies, and deliberate efforts to address stigma. Future research should explore models that support sustained, formalized interagency partnerships to enhance care integration.

## Introduction

Pregnant women who use substances encounter various systems as they navigate through their pregnancy. Studies show that the criminal legal and child welfare services (CWS) systems play pivotal roles in referring women with substance use disorders (SUDs) to treatment [1–4]. Addressing the interplay between these organizations can be an opportunity to improve care coordination and ensure effective treatment for pregnant and parenting women with SUDs. These women often face numerous challenges, including fears of criminal consequences, child custody loss, reduced social and economic support, and coerced treatment [5].

Navigating the criminal legal and CWS agencies is especially complex. A significant proportion (30% to 80%) of pregnant and parenting women with SUDs intersect with the criminal legal and child protective services (CPS) systems [6]. Women in the CPS system often lack awareness of available SUD treatment or have misconceptions, including that they will lose custody of their children, making it difficult for them to access treatment [7]. The criminal legal system serves as one of the primary referral sources for pregnant and parenting women seeking SUD treatment. Yet, those referred by the criminal legal system are less likely to receive evidence-based treatment, such as medication for opioid use disorder, due to stigma, limited resources, policy restrictions, and inadequate integration between legal and healthcare systems [2,8]. The landscape for addressing SUDs among pregnant and parenting women is evolving, with a growing recognition of the need for collaborative and integrated approaches [9–11]. Still, many systems remain fragmented. In this paper, we identify and analyze the barriers and facilitators of inter-organizational collaboration in addressing SUDs among pregnant and parenting women.

Coordination can occur at multiple levels. At the organizational level, coordination involves the linking, meshing, synchronization, or alignment of actions, and bringing contributions together [12]. At the individual level, care coordination refers to the “deliberate organization of patient care activities between two or more participants (including the patient) involved in a patient’s care to facilitate the appropriate delivery of healthcare services” [13].

Effective care coordination has the potential to enhance health outcomes for both parenting people and their children [2,14–16]. Patient-centered, integrated services can mitigate unnecessary duplication and inefficiency, and promote tailored, need-based care for pregnant and parenting women. However, a key challenge in achieving this coordination across agencies is the dearth of research on organizational dynamics, interorganizational interaction, and barriers to integrated care. Analyzing these dynamics is critical for state and federal agencies, stakeholders, and policymakers to attain their goals for quality of care, making it an imperative area for research.

The study questions were guided by the interorganizational relationships framework [17], which identifies key factors that promote and inhibit effective collaboration, including the need to balance competition and cooperation. Daft’s interorganizational relationships framework underscores the importance of trust, shared goals, and clear communication in fostering sustainable collaboration across healthcare, social service, and community organizations serving diverse communities. It highlights how leadership, openness, and flexibility support coordination amid logistical and political challenges. By gaining insight into interorganizational practices and potential barriers to working together across SUD, criminal legal, CWS, and other healthcare organizations that care for pregnant and parenting women, this paper aims to advance understanding of how to build collaborative interorganizational relationships and care coordination practices for a population that often falls through the cracks of siloed systems.

## Materials and methods

### Study design and population

This qualitative study explored dynamics among organizations involved in caring for pregnant and parenting women with SUDs. We recruited participants from various sectors, including criminal legal, CWS, community-based care organizations (CBOs), health agencies, SUD treatment, and other healthcare settings that have experience with care coordination for pregnant and/or parenting women using substances in New York State. Eligible participants were aged 18 or older and employed at relevant organizations. Potential interview participants were identified using publicly available governmental and non-governmental databases and websites, professional networks, and recommendations from participants who had already completed interviews. To capture the range of organizations and activities related to pregnant and parenting women, potential participants were purposively sampled based on job title, institutional affiliation, geography, and the level of involvement working with pregnant and parenting women. We also used snowball sampling, asking participants to refer potential contacts. Potential participants were contacted through email outreach using e-Flyers that indicated the eligibility criteria. Participants were eligible if they were currently employed in the criminal justice system, child welfare services, substance use treatment, or prenatal/postpartum care, and had direct experience working with pregnant or parenting women who use drugs or have a substance use disorder. This criterion ensured the inclusion of professionals with relevant, practice-based insights across key service systems.

### Ethics statement

Prior to conducting interviews, trained research coordinators conducted eligibility screening and obtained verbal informed consent. At the start of each interview, the interviewer used a verbal consent script to explain the study’s purpose, risks, and benefits, address participant questions, and obtained verbal consent for participation and audio recording. We preferred verbal consent to reduce participant burden and support informed decision-making. Interviewers documented verbal consent in accordance with IRB-approved procedures. This process, including our request for a waiver of documentation of consent for obtaining verbal consent, was approved by the NYU Langone Health’s IRB (Protocol #i22-00053).

### Data collection

Between April 2022 and April 2023, trained interviewers (SC, EK, CK) conducted one-on-one semi-structured interviews via Zoom. The semi-structured interview guide explored organizational priorities and culture; experiences coordinating care for pregnant and parenting women with SUDs in New York State; and factors in the external environment of the participating organizations (e.g., system-level policies or priorities for resource allocation and relative priority of coordinating care for pregnant and parenting women) (S1 Fille). Probing questions were used to encourage participants to provide detailed accounts. Interviews, which lasted approximately 45–60 min, were audio-recorded and professionally transcribed. At the end of the interviews, brief surveys collected data on socio-demographics and professional characteristics (e.g., self-reported age, sex, gender, race/ethnicity, current position, time at organization and in current position). After the interview, participants were asked if they could recommend women who met the study objectives and were willing to participate. Participants received $30 e-gift cards for their time.

### Data analysis

A summary was created immediately after each interview and reviewed with the research team during biweekly regular study meetings. Participants were recruited until thematic saturation was reached. Summaries allowed for high-level analysis during ongoing data collection, facilitated codebook development using the interorganizational framework, and reduced the number of initial codes requiring re-review and re-coding of transcripts during data analysis [18]. Thematic analysis was used to analyze and code the transcripts [19] (S1-S2 File). This analysis aimed to uncover patterns of interaction, collaboration, and potential areas for improvement within and across stakeholder groups. Dedoose software was used to code transcripts [20]. Each transcript was coded by at least two independent coordinators (SC, EK, IVD, JO). Coding disagreements were resolved through discussion. A continuous and iterative process of coding, categorizing, and reviewing the raw data was used to reflect on the analysis at various points and make revisions to the codebook.

### Study sample

30 participants were interviewed for this study. Participants were mostly female (90%), and the average age of participants across all groups was 45 years. Participants were affiliated with SUD treatment and other healthcare service providers (n = 10), CWS providers (n = 10), criminal legal stakeholders (n = 6), and other CBOs (n = 4), as illustrated in Table 1. SUD treatment and other healthcare service providers included participants working at both hospital and non-hospital-based service providers. CWS providers included participants affiliated with or contracted by the Office of Children and Family Services (OCFS)/Department of Social Services (DSS) or the Administration for Children’s Services (ACS). Criminal legal stakeholders included participants affiliated with different areas of the legal system, such as courts and correctional services. Non-Hispanic White participants were the most prevalent (70%), followed by non-Hispanic Black participants (13.3%), non-Hispanic Asian participants (10%), and Hispanic participants (6.7%).

**Table 1:** Participant Demographics.

Demographics	SUD Treatment Service Providers and Other Healthcare Systems (n = 10)^a^	Child Welfare Service Providers (n = 10)^a^	Criminal Justice/Legal System (n = 6)^a^	Other CBOs (n = 4)^a^	Total (n = 30)^a^
**Sex**					
Male	0 (0%)	1 (10%)	2 (33.3%)	0 (0%)	3 (10%)
Female	10 (100%)	9 (90%)	4 (66.7%)	4 (100%)	27 (90%)
**Average Age**	36	48	49	49	45
**Race/ethnicity**					
Non-Hispanic Black	2 (20%)	1 (10%)	1 (16.7%)	0 (0%)	4 (13.3%)
Non-Hispanic White	6 (60%)	7 (70%)	4 (66.7%)	4 (100%)	21 (70%)
Non-Hispanic Asian	2 (20%)	0 (0%)	1 (16.7%)	0 (0%)	3 (10%)
Hispanic	0 (0%)	2 (20%)	0 (0%)	0 (0%)	2 (6.7%)

^a^Percentages and sample sizes (N) are provided for each demographic category.

## Results

A wide range of roles contribute to supporting pregnant and parenting women with SUD, each bringing distinct expertise. Coordinators and Case Workers manage care and system navigation; Social Workers offer counseling and advocacy; Clinicians provide medical and behavioral health care; Judges influence legal pathways and support alternatives to incarceration; and Leadership drives program strategy and interagency collaboration. The S3 File lists the diverse participants’ roles involved in caring for pregnant women, even as many shared overlapping responsibilities.

This section highlights the key themes and subthemes identified in the multilevel analysis, centered on the experiences of professionals working with pregnant and parenting women with SUDs (Table 2). The results are categorized into four main themes: navigating complex needs and services; interorganizational arrangements and relationships; organizational-level facilitators and barriers; individual (staff) level facilitators and barriers.

**Table 2:** Facilitators and Barriers to Collaboration Between Agencies that Work with Pregnant and Parenting Women Who have SUDs.

	Facilitators	Barriers
Organizational Level	Formal collaborative efforts (regional and community collaborations) Embedding health professionals within CPS/CWS Alignment between individual staff and organizational leadership Longstanding nature of successful collaborations	Lack of resources including organizational structures to support formal collaborations Knowledge gaps between different areas (e.g., SUDs, child welfare, legal procedures) Staffing problems (high turnover and understaffing) Overwhelming workloads due to external factors (e.g., COVID-19, staff burnout)
Individual Level	Informal, individual collaborations (networking, word of mouth, grassroots outreach) Leveraging individual relationships to overcome system-level barriers Physical proximity between collaborators, ease of communication Proactive outreach from SUD providers to perinatal staff at birthing hospitals Having the right people with genuine care for the work	Vulnerability and lack of sustainability in individual efforts Protective attitudes towards time and resources Unwillingness to try new procedures or collaborative efforts Prioritizing individual program needs over client needs

Each theme addresses different important facets of service provision. Notable factors include the importance of trust-building, effective communication, and the need for better resource allocation at both organizational and individual levels. These factors are critical for understanding the nuances of service delivery across different agencies.

### Navigating complex needs and services

Participants described clients as having complex needs, including those related to pregnancy/parenting, substance use and polysubstance use, and legal involvement. In addition, clients often faced other social and health challenges, such as adverse childhood experiences, food insecurity, housing instability, trauma, mental health conditions, domestic violence, and employment issues. These overlapping needs make coordinated care and collaboration across agencies salient.

“*A lot of the problems that come to me are the system level problems. For example, homeless; we have a huge increase in homelessness. The other one we’re facing is with mental health; that’s probably another big one with regards especially to juveniles currently. Other agencies may not like what we’re doing and, you know, are voicing that, and we’re trying to work through that. I have involvement in a lot of the legal cases that come through as far as protective removals, terminations of parental rights, less so with child support because they don’t, it’s a more standard type of process. That’s kind of a nutshell, I guess*.” (County Leadership in CWS)

“*There’s definitely I’d say – out of every batch of participants – there’s also probably at least one person who’s having housing issues or employment issues. We’ve had a fair amount of people who have intimate partner violence. A lot of, yeah, and all of it exacerbated by there being a baby to take care of.”* (Clinician working in a SUD Treatment Program within a large medical system)

“*They have trauma. That’s also another thing we’re measuring. We have a fairly high rate – because I’m just looking at what the ACES and the TSE-40 scales that we’re using – it’s a fairly higher rate of that. So, they want to just have the mental healthcare. They’re aware enough to know that they’re using substances because something happened in their past*.” (Clinical Director working in a SUD Treatment Program within a large medical system)

### Interorganizational arrangements and relationships

Many organizations relied on referral networks to facilitate access to services. Healthcare providers referred pregnant and parenting women with SUDs to specialized addiction treatment centers, social services, or mental health professionals. Courts often mandated treatment and required paperwork demonstrating attendance and adherence to treatment services. While these networks were useful in ensuring women accessed necessary services, they were often limited by lack of follow-up and inconsistent communication between organizations, leading to fragmented care. Another form of collaboration involved case management teams that coordinated care across multiple organizations. These teams were often led by a designated case manager who facilitated communication between healthcare providers, social services, and other community-based organizations.

Staff within healthcare systems often had social workers or “coordinators” who assisted with navigating not only health services, but also social and legal services. While close collaboration with CPS often proved beneficial for patients, staff members frequently needed to maintain some distance from CPS when working with clients.

*“[Even though we work with CPS] I try really hard to gain [clients’] trust right away. I’ll usually tell them “Our goal here is to help you. We’re not associated with CPS in any way...I usually let them know that right up front*.” (A staff in a healthcare system)

Another notable type of arrangement described by respondents was that between SUD treatment providers and maternity hospitals. In these examples, SUD clinics were formally affiliated with the hospitals at which their pregnant clients and other women with SUDs would give birth. While the affiliation is formal, the collaborative efforts themselves were not codified and were reliant on efforts initiated by the SUD side of the relationship. In this type of collaboration, SUD treatment providers proactively reached out to perinatal staff to provide education about substance use and SUDs in pregnancy and to advocate for individual clients to improve care for patients, decrease stigma, and prevent CPS involvement:

“*Sometimes, I think some patients don’t necessarily want us to talk to their OB provider because of stigma and things like that. But I mean, it’s definitely something – especially, I think, as a birthing hospital, we really have been encouraging staff to talk to the patient about the value because I really think this could actually be helpful to the patient – especially if they’re in treatment, they’re doing good, and they have this plan of safe care. And we can talk to maybe a care coordinator at the hospital and say, “Yes, this is somebody who’s been in care with us,” and I think it could potentially avoid some unnecessary CPS calls, for instance*.” (A leader in a SUD treatment service)

Overall, this theme centers on trust-building and stigma reduction in healthcare and social services for pregnant and parenting women with SUD. Providers emphasize the importance of clarifying their roles to patients, separating themselves from CPS to foster trust and encourage collaboration. By reassuring patients that their goal is supportive care, not punitive action, professionals help mitigate stigma.

“*I always identify myself as “I am not CPS. I am a nurse and I’m here to make sure that you have your services in place. Is your baby developing according to the milestones?” So, there’s a very, very different flavor...Inevitably there is an immediate trust in the nurse. So, that’s what I do. I am a public health nurse, but I have a co-location with CPS*.” (A nursing staff (contractor) at CWS)

### Organizational level facilitators and barriers

At the organizational level, having resources (e.g., grant funding, infrastructure, personnel) that facilitate formal collaborative efforts can significantly improve the coordination of care for pregnant and parenting women with SUDs. Another organizational level facilitator is embedding healthcare professionals within CPS or CWS to enhance service delivery. For example, nurses working alongside CPS staff noted that their physical presence within the CPS office facilitated collaboration and increased referrals, ensuring that clients received the appropriate care (e.g., “had I not been there, [CPS workers] probably would not have been reminded that I might be able to do something for him [client]”).

Next, ensuring the alignment between individual staff members and organizational leaders is crucial for fostering effective collaboration. One leader emphasized that successful partnerships arise when service providers share a common goal—what is in the best interest of the family, not the interests of the providers or their organizations. This family-centered approach requires healthcare professionals to align their treatment and support strategies with the goals of the families they serve, overcoming the biases and traumas that service providers may bring to their work. By collaborating in ways that prioritize families’ needs, professionals can create more successful and sustainable outcomes.

“*I think when people can come together and align with what is supposed to be in the best interests of each family, not in the best interests of us. As service providers...it’s not about us. In social services we, you know, and this is unfortunate...we come in ourselves with our own traumas, our own stereotypes, our own biases. And so, you know that can sometimes also be a barrier, but it does get in the way. And so, I think that whenever you can collaborate with somebody and those interests in that work and that treatment and support is aligning with what’s in the best interests of the family and letting that family guide us is really what I think is the most successful and have been the most successful situations*.” (A staff in CWS)

However, several barriers complicate formal collaboration. A lack of resources and organizational structures to support these efforts can hinder progress as organizations struggle to coordinate care with limited personnel and funding. Staffing issues, including high turnover and understaffing, exacerbate this challenge; organizations cannot always maintain the necessary workforce to ensure continuous care.

The impact of external factors, such as COVID-19 and staff burnout, further overwhelms service providers, leading to decreased capacity for meaningful collaboration. These challenges also highlight knowledge gaps among specialists in SUD treatment, child welfare, and legal procedures, which can create misalignment in goals and hinder effective communication between organizations. As one clinician noted, for example, when workers are focused on completing paperwork, the broader picture of client engagement and motivation often gets overlooked, which could be better addressed with more frequent and open communication.

“*My general impression is that ACS workers are usually very busy. So, yeah – the thing they want from us, the treatment provider, usually is attendance and view tox results. However, we do feel like there are really a lot more just beyond some of the factual things…like… how the client attendance because…really, that shows some level engagement, but that doesn’t tell a lot about the client engagement – or the motivation of change in treatment. So, I just hope the ACS worker can…yeah, communicate with us more often instead of just wanting some letter…from us*.” (Clinical Coordinator in SUD treatment)

Despite these challenges, longstanding collaborations have proven successful. For example, grant-funded programs like the Regional Partnership Grant, which focuses on supporting new and expecting mothers with a history of substance use, have contributed to improved outcomes by fostering collaborative partnerships across disciplines. These formal efforts can help promote comprehensive, coordinated care for women affected by SUDs, although more organizational support and resources are necessary to maximize their potential.

### Individual (staff) level facilitators and barriers

Collaboration often takes the form of individual initiatives where staff leverage their relationships to connect clients with needed services. For example, a clinician working in SUD treatment noted the importance of personal outreach, stating, *“I mean well we don’t have an official partnership with our programs at the agency, but they will refer to us whenever there’s a pregnant woman in the program. They’ll definitely call us to screen them out.”* (Clinician, SUD treatment in a Hospital Setting)

The presence of individuals who are dedicated to the work can make a substantial difference. One staff member from the criminal legal system remarked, *“When we brought in this coordinator who really knew what she was doing, it made all the difference in the world because she was able to connect with these women, and she found these programs, mother and child programs.”*

However, these individual efforts often face limitations in terms of resources and organizational support. A leader in the healthcare system emphasized that success often relies on personal relationships and time, stating, *“Time is on your side in a place like this – networking, knowing your personnel, and you can pretty much get anything... Pretty much, I’m able to get everything I need, and what I need to get done, it’ll get done.”* Despite the importance of networking and personal connections, the lack of structural support can undermine these efforts.

A staff member from child welfare noted, *“Supervisors or managers are really pushing caseworkers or other institutions to really collaborate...I mean it can be daunting. Of course, it’s great that people are talking, but...we can talk all we want, but if there’s not the money and then the resources, that’s what’s holding [us] back.”*

Lastly, a critical challenge identified was the difficulty in overcoming knowledge gaps between specialists in different service areas. A staff member in a community-based organization pointed out, *“There’s definitely a misunderstanding between those who work in substance use disorder and those who work in...mental health, child welfare, other areas. I feel like there’s people who understand addiction treatment [but] don’t understand mental health services or child welfare services.”* These gaps in understanding make collaboration challenging and can hinder the effectiveness of efforts, even when individuals are willing to work together.

## Discussion

Our study provides insights into the nature of relationships between organizations and systems that work with pregnant and parenting women with SUDs. Our findings underscore the complex dynamics of collaboration at the intersection of SUD treatment, CWS, criminal legal, and other CBOs, at both the organizational and individual levels.

The participants highlight the complex and often fragmented nature of support systems for pregnant and parenting women with SUD. Multiple types of partnerships, arrangements, and innovations have emerged to help guide women through the criminal legal, CWS, and SUD treatment systems. However, coordination efforts often begin only after a triggering event—such as involvement in the court system, contact with CPS (frequently post-delivery), discovery of pregnancy, or admission to SUD treatment. This reactive approach can delay access to comprehensive care and support. It also reflects broader systemic challenges, including limited integration across sectors and a lack of early intervention pathways.

At the organizational level, formal collaborative efforts, such as regional and community collaborations, reflect Daft’s emphasis on formal mechanisms that structure interorganizational cooperation and help address systemic barriers. These collaborations are often more impactful when health professionals are embedded within CPS and CWS, which fosters communication and interdependence, key relational factors in Daft’s framework that promote coordinated approach to client care. Moreover, alignment between individual staff members and organizational leadership and openness in creating an environment that supports and sustains collaboration. Longstanding partnerships built on trust and shared goals provide the relational foundation necessary for continued success, underscoring the interplay of relational and organizational characteristics central to Daft’s theory.

At the individual level, informal collaborations—such as networking, word-of-mouth referrals, and proactive outreach—are central to overcoming barriers and ensuring clients receive the necessary services. Personal relationships often drive these collaborations, bridging gaps between siloed systems and facilitating critical connections for clients. However, these efforts are vulnerable to limitations in staffing, time, and resources, which can undermine their long-term sustainability and impact. Relying on these informal networks raises concerns about sustainability and the risk of losing momentum with staff burnout and turnover. There is a need to develop more robust organizational structures to support and sustain these efforts.

These findings align with resource dependence theory [21], which explains how organizations’ actions are shaped by their reliance on external resources and the interdependencies that arise from this dependence. The fragmentation of services often exacerbates this issue, with the responsibility of managing complex problems falling heavily on individual case managers and coordinators, who are crucial in navigating and coordinating care across multiple service providers. Addressing resource constraints and staffing issues is crucial. Policymakers should consider ways to increase funding and support for services that ease access to treatment services, recognizing that effective collaboration requires adequate resources. Put differently, it is important to acknowledge that addressing fragmentation is not solely the responsibility of individual staff members, but requires a collective effort from various stakeholders. Cross-sector training programs could help bridge knowledge gaps and foster mutual understanding between different service providers. Such training could be incorporated into professional development requirements for those working in SUD treatment, CWS, criminal legal systems, and related fields.

Our findings amplify results from prior work, especially the importance of both informal and formal collaborations, while also highlighting the need for improvement in these collaborative efforts [22]. For example, a recent paper mentions the nature of organizational siloes in caring for pregnant and parenting women who have SUDs and the need for collaborative approaches that are non-punitive [23]. Non-punitive approaches focus on support and recovery, rather than punishment, which is essential for reducing the stigma and fear that often prevent women from seeking help. By creating systems of care that prioritize empathy, understanding, and cooperation across sectors—especially healthcare, CWS, and community organizations—providers can offer more effective and compassionate care, ensuring that pregnant and parenting women with SUDs receive the treatment and support they need to thrive.

Recognizing this, there are initiatives aimed at formally institutionalizing support for these individual efforts. Perinatal peer recovery coaches— women with lived experience in substance use recovery—can play a role in enhancing engagement and retention in SUD treatment programs [24,25]. One study demonstrates that peer recovery coaches can be highly effective in providing guidance, advocacy, and emotional support for parents involved with CPS [26]. These studies indicate that these support roles not only help parents comply with CPS requirements but also foster long-term recovery and family stability. However, challenges remain in hiring and retaining these coaches, especially those from communities of color, due to low salaries, inflexible policies, and the complexities of their personal circumstances [24]. Regardless, prior work points to the importance of hiring peer coaches or integrating women with lived experience into formal structures to improve health care access and outcomes for marginalized communities.

Our study also revealed that working in isolation can inadvertently give rise to stigma, particularly in the context of SUD treatment and CWS. In our findings, a healthcare provider made it explicitly clear to a patient that they were separate from CPS, which raises important questions about the role of collaboration in building or eroding trust. This underscores the need for increased integration and collaboration to combat stigmatization and improve outcomes for families affected by SUDs. On the one hand, stronger collaboration between healthcare providers and CPS could help reduce the “us vs. them” mentality, making patients feel that they have a unified support system rather than separate entities with competing interests. However, this same collaboration could also have the opposite effect—when patients perceive that healthcare providers are too closely aligned with CPS, their trust can erode and discourage them from engaging with medical care providers. Future research should explore how different approaches to collaboration impact trust, particularly among populations with historical mistrust of child welfare and healthcare systems.

Based on our findings and grounded in Daft’s interorganizational relationships framework [17], several concrete strategies can support stronger collaboration across agencies serving pregnant and parenting women with substance use disorders. First, establishing regular, structured cross-agency meetings or communication channels can enhance trust, communication, and alignment around shared goals—key relational factors that underpin successful partnerships. Second, developing formal protocols and memoranda of understanding can clarify roles and responsibilities, providing the formal mechanisms necessary to reduce ambiguity and foster interdependence. Third, investing in embedded liaison roles or cross-trained staff can bridge organizational boundaries by leveraging experienced, committed individuals who facilitate coordination and continuity of care. Furthermore, cultivating organizational openness and flexibility enables agencies to adapt workflows and share information more effectively in response to evolving needs. Finally, recognizing and managing the balance between cooperation and competition—Daft’s concept of cooperation and competition—allows organizations to maintain their distinct missions while working collaboratively toward shared outcomes.

Future research should explore the outcomes of different collaborative models to identify the most effective approaches for improving care coordination and service delivery. Studies should investigate how different models impact client outcomes, including substance use recovery, mental health, and overall family well-being. Research should focus on strategies for overcoming identified barriers, such as addressing resource gaps, improving interagency communication, and enhancing staff training. By examining these factors, future studies can provide actionable recommendations for enhancing collaboration and ensuring better outcomes for women with substance use disorders and complex needs. Additionally, studies that include client perspectives on collaboration in SUD treatment, criminal legal systems, and CWS would be valuable. Finally, organizational cultures that prioritize client outcomes over arbitrary organizational measures should be fostered. This may require rethinking or introducing performance measures and incentives in these sectors.

Our study has limitations. Our use of purposive sampling may introduce bias in participant selection, potentially affecting the representativeness of the sample. Second, because our study was conducted within New York State, the generalizability of our findings may be limited, particularly when considering different state or regional contexts with varying systems, resources, and policies. Finally, the study’s qualitative nature means that our findings are based on participant self-report, which can be subject to bias or limitations in recall. These factors should be considered when interpreting our findings, applying the results to broader contexts, and making policy and practice recommendations.

## Conclusion

In conclusion, while both informal and formal collaborations play crucial roles in enhancing service delivery for pregnant and parenting women with SUDs and their families, addressing systemic barriers and fostering a culture of collaboration remain essential for improving outcomes. By building on the strengths of existing collaborative efforts and addressing the identified challenges, we can work towards more integrated and effective care for this vulnerable population.

## Supporting information

S1 File Interview Guide. (DOCX)

S2 File Example Codebook. (DOCX)

S3 File Interviewee Roles. (DOCX)
