# Supplementary material for: Understanding barriers and facilitators of inter-organizational dynamics in addressing substance use disorder among pregnant and parenting women
Source: PLoS One. 2025 Nov 12;20(11):e0336029. doi: 10.1371/journal.pone.0336029 (PMC12611144; doi:10.1371/journal.pone.0336029)
Supplement: S1 File — (DOCX) [file pone.0336029.s001.docx]

**S1 File. Interview Guide**

**Interviewer ID _________**

1. **Interview Date (DD/MM/YYYY) __________________**
2. **Time Start _______**
3. **Time End _______**
4. **Setting (circle one)** (Criminal Justice / Child Protective Services / Foster Care System / Addiction Treatment / Other: ___________)
5. **Organization/Agency name __________________**

**Section 1: Participant Background**

1. **We would like to know a bit about your current role.**
2. **What is your title?**

**____________________**

1. **How long have you been with __[name of institution]__?**

**____________________**

1. **How long have you been in this role?**

**____________________**

1. **Have you held a similar role at a different institution?**

**____________________**

1. **Have you received any training in working with caregivers, women, and children? If so, please describe this training.**
2. **To get a sense of your day-to-day work, walk me through what you do on a regular basis.**

**Probe(s):**

1. What activities are you engaged in?
2. With whom are you working closely?
3. How has the way you do your job been affected by COVID-19?
4. **Given that your role at the __[institution name]__ entails ___[description]___, what are your current priorities?**

**Probe(s):**

- 1. How do you think these align – or not – with [coordinating care/offering services] for women with SUD?

1. **How do you see addressing pregnant people and parenting women’s needs as part of your job?**

**Probe(s):**

- 1. How do you see your role as impacting SUD treatment access/use for pregnant/parenting women with SUD?
  2. Are you directly involved in any initiatives or programs aimed directly at pregnant/parenting women with SUD?
  3. Are you or is [name of the institution] directly involved in screening SUD? If so, could you describe the screening process?
  4. How have your priorities shifted during your time at [name of institution]?

1. **Could you tell me about some of the service or treatment options that are currently available for pregnant and parenting women with SUD?**

**Probe(s):**

1. How would you describe the organizational culture? How does this impact how you work with pregnant/parenting persons/women?
2. What is your organizational approach or philosophy when serving pregnant/parenting persons/women?
   1. How are priorities set and new initiatives identified? What does that process look like?
3. What are the current initiatives that target pregnant and/or parenting women with children?
   - 1. How have initiatives changed with COVID-19?
   1. What are some changes you would like to see to improve treatment for pregnant and/or parenting women with SUD or their children?
4. **How do you define and measure success in the treatment of pregnant/parenting women with SUD?**

**Probe(s):**

- 1. How has COVID-19 affected this?

1. **Which organizations do you work with to help women with SUD? Please describe the care coordination process for me.**

**Probe(s):**

- 1. Where [what services] do you refer patients out to?
  2. How often do you refer patients out to other services (i.e., treatment, social services, foster care, housing, employment services, community-based services)?
  3. How do you decide if you need to refer peoples' care out?
     1. Who makes the determination that a child/parent is at risk?
  4. What is the process for referrals like? How do you follow up?
  5. How do you know if referrals have been completed?
  6. What was the process like for establishing a referral workflow?
  7. How are these relationships/partnerships going?

1. **In thinking about working together with other agencies or organizations to help pregnant and parenting women with SUD, what do you see as the biggest barriers (if time, as for facilitators) for you and/or the organization?** (i.e., Perceived barriers of coordinating care for pregnant and parenting women and the organization)

**Probe(s):**

- 1. How do you [How does your organization] communicate? How do they share information?

1. **In thinking about working together with other agencies or organizations to help pregnant and parenting women with SUD, what do you see as the biggest facilitators for you and/or the organization?** (i.e., Perceived facilitators of coordinating care for pregnant and parenting women and the organization)
2. **The Family First Prevention Services Act (Family First) was signed into law in 2018. It provides opportunities for child welfare agencies and their partners to improve the lives of children and families touched by the child welfare system. Is [name of the institution] involved in implementing services as part of this reform?** (examples include SUD treatment, parent skill-based programs including parenting skills training, parent education, counseling, or mental health services)
3. **What would you improve in *your particular scope of work* coordinating care for pregnant and/or parenting women/persons?**
4. **What would you improve in *your organization* for pregnant and/or parenting women/persons?**
5. **In addition to what we have covered, what else is important for me to know?**
