# Supplementary material for: Understanding barriers and facilitators of inter-organizational dynamics in addressing substance use disorder among pregnant and parenting women
Source: PLoS One. 2025 Nov 12;20(11):e0336029. doi: 10.1371/journal.pone.0336029 (PMC12611144; doi:10.1371/journal.pone.0336029)
Supplement: S2 File — (DOCX) [file pone.0336029.s002.docx]

S2 File. Example Codebook

| **Code name** | **Parent Code/Child Code** | **Definition** | **Example(s)** |
| --- | --- | --- | --- |
| **Organization relationship** | P | Inter organizational relationships; relatively enduring resources transactions, flows, and linkages among two or more organizations. | Court receives referrals from ACS |
| Competitive | C | Adversarial relationships; where two or more parties strive for a common goal which cannot be shared | Bidding |
| Cooperative | C | Collaboration/cooperation; working or acting together for common, mutual, or some underlying benefit, as opposed to working in competition for selfish benefit | Formal cooperation; Contracts  Informal cooperation; agreement, norm, tradition |
| **Organization type** | P |  |  |
| Child protective services (CPS) | C |  |  |
| Administration for Children's Services (ACS) | C |  |  |
| Drug court | C |  |  |
| Family court | C |  |  |
| Opioid court | C |  |  |
| SUD treatment | C |  |  |
| Hospital/OB/GYN | C |  |  |
| Non-profit organizations | C |  |  |
| **Goals/missions/roles** | P | Goals/missions of organizations or ambiguity in goals/roles  [motivation]; Self-interest? | “our goal is to help families re-unite” |
| **Services provided (Other than care coordination)** | P | Treatment, health care services, home visits, etc | Counseling, parenting classes, yoga classes, food, etc |
| **Cooperation** | P | Organizations share info and may work together but do not coordinate care together (do not plan care together, but may talk about what each of them is doing) |  |
| **Care coordination** | P | Deliberately planning and organizing patient care activities and sharing information among all of the participants concerned, or mention of a care model; joint discussions  *how extensive is it? Shared decision making? | Having case conferences, Calling activities, Following-up after a patient visit |
| Organizational facilitators/barriers | C | Organizational factors that help/do not help coordinating care for pregnant/parenting women | Organizational structure/process/  Communication styles/staffing/workload/  Supervision/leaderships/ culture/trust/value/  Infrastructure/resources |
| Environmental, community facilitators/barriers | C | Facilitators/barriers external to the organization that help/do not help coordinating care for pregnant/parenting women | Environmental influences, community context, public policy |
| Patient level factors | C | Patient or family level facilitators/barriers that help/do not help coordinating care for pregnant/parenting women | Sociodemographics, access to resources (e.g., housing) |
| **Care duplication or ignorance** | P | Organizations seem to be doing similar things with/without consulting each other |  |
| **Key quote** | P | Flagging quotes that can be useful for explaining care coordination efforts/choosing best quotes for manuscript or presentations |  |
| **Innovation** | P | a new method, idea, intervention to promote coordination efforts across agencies | Telehealth during COVID |
| **Gaps** | P | Identified gap(s) in care coordination or providing care for pregnant/parenting women | “Transportation is something we cannot provide at the moment” |
| **Harm reduction** | P | strategies that includes safer use, managed use, abstinence, meeting people who use drugs “where they're at,” and addressing conditions of use along with the use itself |  |
| **Race/ethnic disparities** | P | Mentions racial/ethnic differences/disparities in clientele and efforts to reduce disparities/inequities |  |
