# Supplementary figures and images for: Understanding barriers and facilitators of inter-organizational dynamics in addressing substance use disorder among pregnant and parenting women
Source: PLoS One. 2025 Nov 12;20(11):e0336029. doi: 10.1371/journal.pone.0336029 (PMC12611144; doi:10.1371/journal.pone.0336029)

S3 File. Interviewee Roles


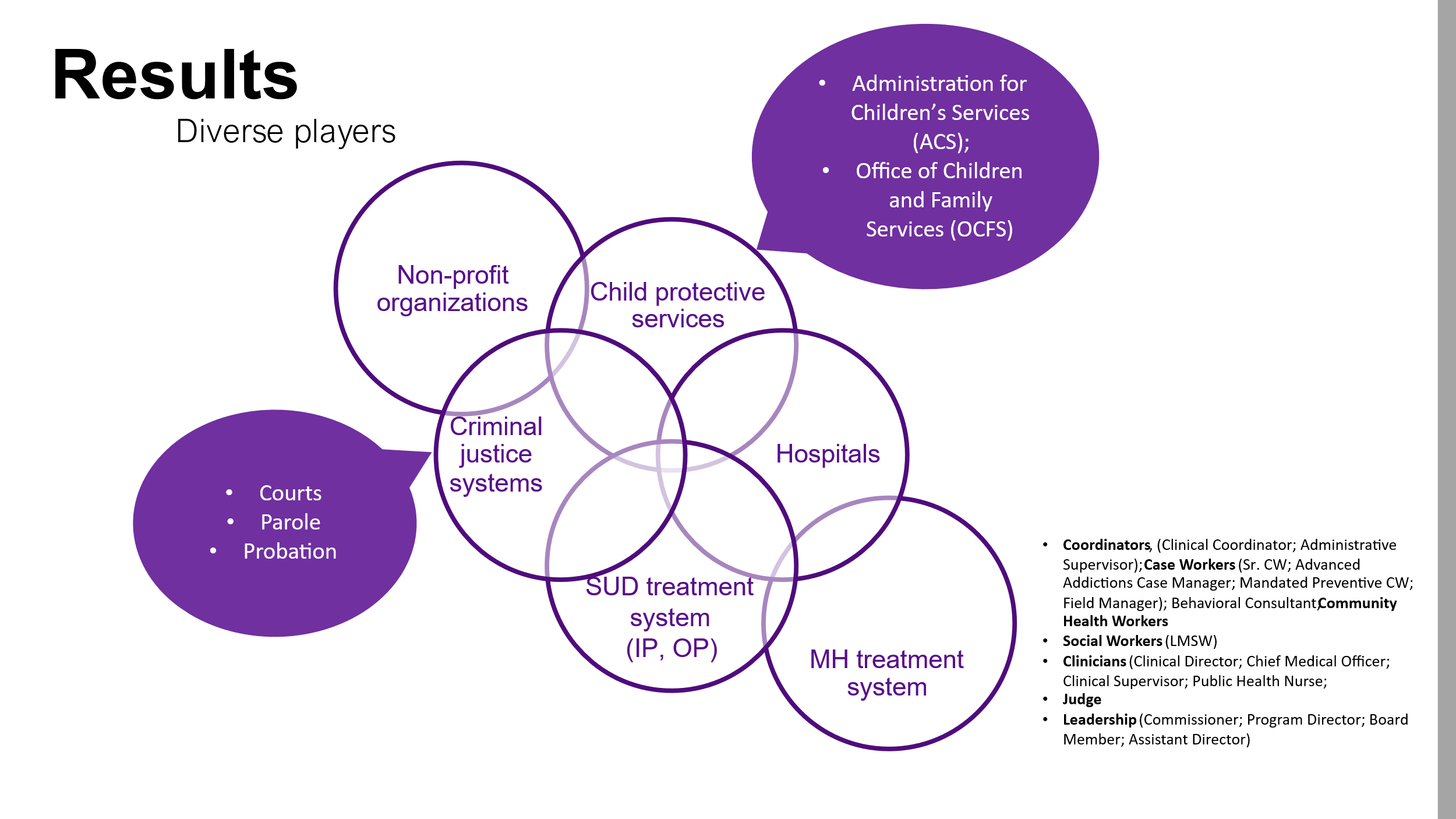

Supplement: S3 File — (DOCX) [file pone.0336029.s003.docx]
